# H-Type Indices With Applications in Chemometrics: h-Accuracy Index for Evaluating and Comparing Errors in Analytical Chemistry

**DOI:** 10.1155/2024/1580359

**Published:** 2024-10-16

**Authors:** Lu Xu, Xihui Bian, Qin Yang

**Affiliations:** ^1^Department of Nursing and Health, School of Sports and Health Science, Tongren University, Tongren 554300, China; ^2^State Key Laboratory of Separation Membranes and Membrane Processes, School of Chemistry and Chemical Engineering, Tiangong University, Tianjin 300387, China; ^3^Department of Optoelectronics, School of Physics and Optoelectronic Engineering, Yangtze University, Jingzhou 434023, China

**Keywords:** analytical errors, h-accuracy index (HAI), h-index, method evaluation, multivariate calibration

## Abstract

Inspired by the popular h-index in bibliometrics, the h-accuracy index (HAI) was suggested as a new indicator to evaluate and compare errors in analytical chemistry. The HAI simultaneously considers the “trueness” of analytical measurements and the frequency of measurements with high “trueness”. The HAI was defined as if the “trueness” of at most M% of the total measurements is no less than M%, the value of HAI will be M%, where a specific definition of “trueness” was given to compute the HAI. The range of the HAI was between zero and one. The HAI was applied to two examples as a new error index: (1) to evaluate and compare the analytical results by different methods/labs and (2) to evaluate and compare the prediction performances of different multivariate calibration models. Data analysis indicated that the HAI was a reasonable, robust, easy-to-compute, and comprehensive index for evaluating and comparing errors in analytical chemistry. Moreover, the HAI could provide the information about how many measurements are good.

## 1. Introduction

With the rapid development of science and technology, new technologies and methods are constantly emerging in the field of analytical chemistry [1–3]. These new technologies and methods have significant advantages in improving analytical efficiency, reducing costs, and improving detection sensitivity. However, how to fairly and accurately evaluate and compare the analytical results by these different methods has become an urgent problem to be solved [4]. To address this issue, a new evaluation index can be introduced to provide a unified evaluation standard for different methods. The existing evaluation indices are mainly based on measurement errors, including the following aspects. Precision [5] refers to the degree of closeness between results obtained from multiple repeated measurements under the same conditions. High precision means that the variability of the results is small, which can improve the reliability of the measurement results. Accuracy [6] refers to the degree of closeness between the measurement results and the true values. High accuracy means that the measurement results are closer to the true values, which can reduce errors. Considering both precision and accuracy is of great significance in ensuring the reliability of data and improving the quality of research and decision-making [7]. Reproducibility [8] refers to the consistency of results obtained when measured using the same method in different laboratories or at different times. High reproducibility means the stability and reliability of the method. Sensitivity [9] refers to the ability of a method to accurately detect the substance being tested at low concentrations. High sensitivity can improve the detection range of the method. Selectivity [10] refers to the ability of a method to detect specific substances, that is, the ability to only detect target substances in complex samples. High selectivity can reduce interference and improve the accuracy of analytical results. The detection limit [11] refers to the minimum concentration that a method can detect. A low detection limit means that the method has higher sensitivity. The quantification limit [12] refers to the minimum concentration that a method can accurately quantify. Low quantification limit can improve the quantitative ability of the method. Linear range [13] refers to the range within which a method's measurement results show a linear relationship with concentration within a certain concentration range. A wide linear range can improve the applicability of the method. Stability [14] refers to the consistency of the measurement results of a method over a certain period of time. High stability can reduce errors that vary over time.

By introducing these evaluation indices, a comprehensive and objective evaluation and comparison of different analytical chemistry methods can be conducted, providing more valuable references for researchers and industry. Meanwhile, these evaluation indices can also promote technological progress and innovation in the field of analytical chemistry [1]. In analytical chemistry, ensuring the accuracy and reliability of analytical results is crucial [7]. In certain applications, such as environmental monitoring [15], food safety testing [16], and clinical diagnosis [17], the accuracy of analysis results is directly related to people's health and safety. Therefore, understanding the frequency or probability of the analysis results, i.e., the accuracy and reliability of the analysis method, is crucial for evaluating the effectiveness of the analysis method. We may be concerned about the frequency or probability of providing good analysis results, as each failed analysis comes with a certain cost. The purpose of this article is to propose a new analysis evaluation index that compares the errors by different analysis methods or analysts based on the frequency or probability of providing good results.

Academic influence is an essential embodiment of a scholar, an institution, or a country or region's contribution to science and technology [18]. Moreover, academic influence is often accompanied by the incentive mechanism of scholars and institutions, which will in turn play a key role in planning and development of scientific research [19]. Therefore, how to promote the evaluation of academic influence to be more objective and reasonable has always been one of the hot research topics in the field of library science and bibliometrics [20, 21]. Due to the complexity in evaluation of scientific research outputs, traditional criteria are not satisfactory [22, 23] and many scholars devoted themselves to the research of better evaluation indices for academic influence [24–26]. In 2005, Professor Hirsch, a physicist at the University of California, San Diego, creatively proposed a new index, h-index [27], to measure the academic influence of scholars. It not only considers the citations of papers published by a scholar but also considers the number of highly cited papers. Moreover, the calculation method is simple and reasonable. It has aroused strong interest in the whole scientific community in evaluating academic influence [28, 29] and has been regarded as a significant contribution to bibliometrics. Its application soon expanded to evaluating the academic influence of journals, institution, patents, and funds [30–33]. Moreover, many improved versions of h-index and related indices have been proposed and studied [34–36].

As a data analysis method, how can h-index achieve the great popularity and success in bibliometrics? The following features of h-index may shed light on the above question and might also be interesting to a chemometrician:

1. It is very simple and easy to compute
2. It reflects more than one aspect of the data, e.g., the number of citations (strength of a variable) and the number of highly cited papers (the number of objects with high strength) in a single indicator
3. It is not simply an accumulation or average of the data but it is robust against a few outliers

Inspired by the idea of the original h-index, in this work, an h-accuracy index (HAI) was suggested to evaluate and compare the errors of different analytical methods or multivariate calibration models. The HAI was compared with some traditional analytical indices to demonstrate its characteristics and advantages.

## 2. Methods

### 2.1. h-Index in Bibliometrics

In bibliometrics, the definition of h-index was as follows.

**Figure 1:**
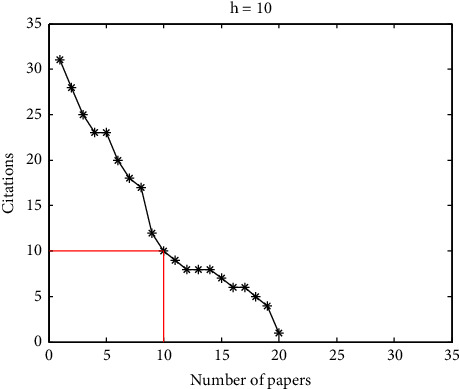
The definition of h-index in bibliometrics.

Suppose a scholar has *N*_*p*_ papers, the value of the h-index will be h if at most h of the *N*_*p*_ papers have at least h citations each and the other (*N*_*p*_ − *h*) papers have ≤ h citations each, where *N*_*p*_ represents the total number of the scholar's published papers and *h* denotes the number of the published papers that have > *h* citations each. In Figure 1, the citations of the 20 papers of a fictional scholar are arranged in a descending order and 10 of the 20 papers has at least 10 citations, while each of the other 10 papers has no more than 10 citations. As a result, the h-index will be 10 in this example. Geometrically, the h-index is the width of the largest square under the curve of citations.

### 2.2. HAI in Analytical Chemistry

The HAI was defined as follows.

For N analytical measurements, if at most M% of the *N* measurements have a “trueness” no less than M%, the HAI of the *N* measurements will be M%.

In this work, a specific “trueness” (*T*) of a single measurement was defined as

(1) $$\begin{array}{c} T_{i}=\max\left\{0,\;1-\left|\frac{x_{i}-x}{x}\right|\right\}, \end{array}$$

where *x*_*i*_ is the value of the *i*th measurement and *x* the reference value.

Some remarks can be made on the HAI. Just like the original h-index could measure the overall citations of several papers, the HAI will measure the overall “trueness” of a series of measurements in the same manner with a single index. The HAI simultaneously considers the “trueness” of measurements and the frequency (in percent) of measurements with high “trueness;” therefore, it might be a reasonable index to reflect the analytical quality or capacity. As for the value of “trueness,” obviously, the closer a measurement is to the reference value, the higher the “trueness” value is.

**Figure 2:**
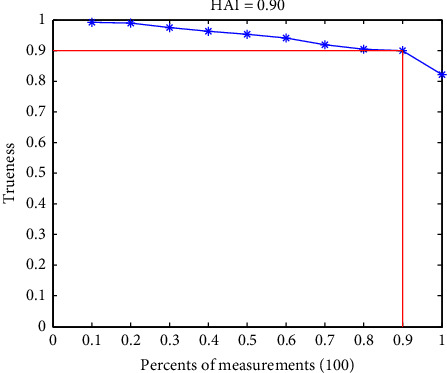
The defnition of h-accuracy index (HAl) as an analytical figure of merit.

To exemplify the computation of the HAI, suppose one has taken 10 analytical measurements and nine of them (90%) have a “trueness” value no less than 0.90, the “trueness” of the 10 measurements is arranged in a descending order and plotted against (one-percentile/100). According the above definition, the HAI of the 10 measurements is 0.9 (Figure 2). Geometrically, the HAI is the width of the largest square under the curve of “trueness.”

### 2.3. Software

The data analysis was performed using MATLAB R2013b (Mathworks, Sherborn, MA, United States of America). The MATLAB codes to compute the HAI can be requested from the corresponding authors.

## 3. Data Analysis and Discussion

### 3.1. Evaluation and Comparison of the Quality of Different Analytical Methods

The analytical results of two analytical methods for 15 test objects with reference values (Table 1) were simulated and used to compare different error indices. For the analytical results in Table 1, the average absolute relative deviation (AARD) of the two methods were 1.4% and 5.0%, respectively, and the root mean square error (RMSE) of the two methods were 0.11 and 0.35, respectively. Both AARD and RMSE indicated that Method 1 was more accurate and had lower analytical errors than the latter. The HAI of the two methods were 0.955 and 0.886, respectively. For Method 1, an HAI value of 0.955 means that at most 95.5% of all the measurements' “trueness” was no less than 0.955. Obviously, a higher HAI corresponded to a better analytical method. In this example, the HAI could obtain the similar results as RMSE and AARD and seemed to be a reasonable index to evaluate an analytical method. Another possible advantage of the HAI might be that the HAI could tell us how many measurements were good, e.g., we knew from the above example that 95.5% of the measurements was good (with a “trueness” no less than 0.955). In some cases, every poor analysis result might come with a certain cost, and we were more concerned about how many measurements were good or satisfactory. The HAI could provide us such information concerning the frequency or probability of providing good analysis results.

**Figure 3:**
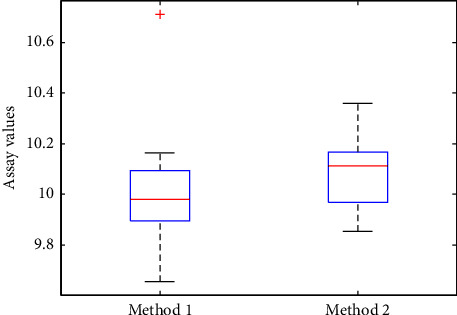
The boxplots of 11 repeated measurements by the two methods.

For the other example as shown in Table 2, two analytical results were simulated with 11 repeated measurements of an object with the reference value of 10.00. As seen from the boxplots (Figure 3), Method 2 seemed to give results higher than Method 1 except that Method 1 had an obvious outlier (10.71) among the 11 measurements. With the outlier, AARD and RMSE of the two methods were 1.7% versus 1.4% (AARD) and 0.26 versus 0.18 (RMSE), respectively. Obviously, the outlier could severely influence the values of AARD and RMSE. Similarly, the HAI values of two methods were 0.936 versus 0.964, indicating the results of Method 2 were better than that of Method 1. By removing the 11th measurements of the two methods, which had the largest deviations from the reference value, the AARD and RMSE of the two methods became 1.2% versus 1.2% (AARD) and 0.15 versus 0.15 (RMSE), respectively. The HAI values of the two methods were both 0.996. With the outliers removed, AARD, RMSE, and HAI gave the similar evaluation results of the two methods. It was demonstrated by the above data analysis that the HAI was a more robust error index than AARD or RMSE.

### 3.2. A New Index to Evaluate Multivariate Calibration Models

In multivariate calibration, the RMSE [37, 38] and the predictive squared correlation coefficient (RMSEP) (*Q*^2^) [39, 40] are two usually used indices to evaluate and compare model prediction ability. In this work, the HAI was compared with RMSE and *Q*^2^ to evaluate model prediction performances of multivariate calibration models including different number of latent variables (LVs). This meat dataset [41] was used for comparison of different indices. The near-infrared transmission (NIT) spectra in the wavelength range of 850–1050 nm (100 channels) were used to calibrate the fat content of finely chopped pure meat. The dataset had 172 training objects and 43 test objects.

**Figure 4:**
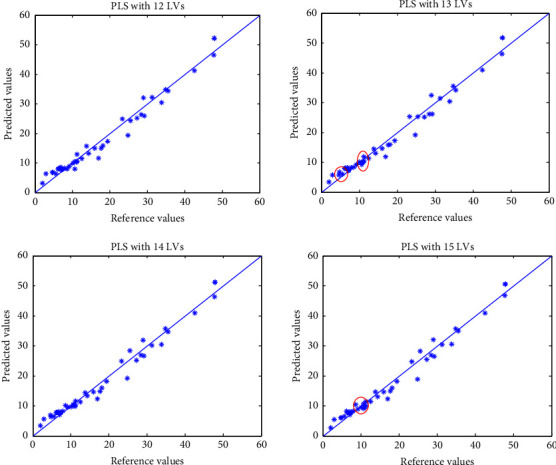
PLS predictions versus reference values for the meat data.

A previous study [42] indicated that PLS models with 14 LVs would be suitable for multivariate calibration. In this work, the RMSEP, *Q*^2^, and HAI values of four PLS models obtained by including 12–15 LVs were computed and compared (Table 3). As seen from Table 3, RMSEP and *Q*^2^ gave consistent results of model comparison, by including more LVs, the model had a lower RMSEP and higher *Q*^2^. The results of HAI were slightly different. The HAI values of different PLS models were 0.779 (12 LVs), 0.798 (13 LVs), 0.807(14 LVs), and 0.806 (15 LVs), respectively. Why did PLS models using 13 LVs and 15 LVs give slightly lower HAI values? This could be explained by the definition of HAI and “trueness,” where deviations of the lower reference values tended to give a lower “trueness” and HAI. The predicted values versus reference values were plotted in Figure 4, where the PLS models with both 13 LVs and 15 LVs seemed to have more deviations of lower reference values ranging from 6 to 15.  Table 4 represents the corresponding reference values, predicted values, and calculated “trueness” values. Generally, the prediction results by using 12–15 LVs were generally fine, and the HAI could give similar evaluations as RMSEP and *Q*^2^. Different from RMSEP and *Q*^2^, which were computed by accumulations and means of all the deviations, the HAI simultaneously considered the deviations and the number of measurements with lower deviations (or a better “trueness”). As discussed in the previous example, the HAI was also a robust index that is less sensitive with a few larger deviations. The results indicated that the HAI could provide a reasonable and useful alternative index to the traditional RMSEP and *Q*^2^ for evaluating multivariate calibration models.

## 4. Conclusions

In this work, inspired by the popular h-index, an HAI was proposed as a new index of analytical errors. The HAI was used to evaluate and compare the results of different analytical methods and multivariate calibration models. Compared with the traditional indices, including AARD, RMSE, RMSEP, and *Q*^2^, the HAI could give the similar results and proved to be a reasonable error index. Moreover, the HAI simultaneously considered the low deviations and the number of measurements with lower deviations and was robust against a few higher deviations. As an error index, the HAI could reflect how many measurements have achieved good results, which was useful when we attached equal importance to the results of each analysis measurement.

## Figures and Tables

**Table 1:** Simulated results obtained by two analytical methods.

Samples	Reference value	Assay value by method 1	Assay value by method 2
1	8.17	8.12	8.74
2	9.34	9.33	9.18
3	2.35	2.30	2.16
4	5.58	5.76	5.40
5	16.42	16.33	16.13
6	2.50	2.42	2.16
7	18.45	18.29	18.14
8	13.47	13.46	13.19
9	12.50	12.60	12.25
10	8.50	8.40	9.21
11	7.99	7.80	7.63
12	2.95	3.09	2.51
13	19.16	19.17	19.32
14	6.56	6.66	6.30
15	14.44	14.41	14.63

**Table 2:** Simulated analytical results of one object with 11 repeats.

	11 repeated measurements^a^		
Method 1	9.94	9.92	10.05
	9.82	9.66	10.10
	9.98	9.89	10.16
	10.09	10.71	

Method 2	9.96	9.99	9.85
	10.12	10.11	10.34
	10.17	10.06	10.15
	9.95	10.36	

^a^The reference assay value was 10.00.

**Table 3:** Prediction results of fat content in meat by partial least squares (PLS) including 12–15 latent variables (LVs).

	RMSEP	**Q** ^2^	HAI
PLS with 12 LVs	2.24	0.971	0.779
PLS with 13 LVs	2.10	0.974	0.798
PLS with 14 LVs	2.01	0.976	0.807
PLS with 15 LVs	1.97	0.977	0.806

Abbreviations: RMSEP = root mean square error of prediction, HAI = h-accuracy index.

**Table 4:** Lower reference values, the corresponding predicted values, and calculated “trueness” values among partial least squares (PLS) with 12–15 latent variables (LVs).

Reference values	Predicted values				Calculated “trueness” values (HAI)			
	PLS with 12 LVs	PLS with 13 LVs	PLS with 14 LVs	PLS with 15 LVs	PLS with 12 LVs	PLS with 13 LVs	PLS with 14 LVs	PLS with 15 LVs
6.20	8.02	7.92	7.79	8.23	0.707	0.722	**0.744**	0.672
6.40	8.11	8.17	7.79	7.59	0.733	0.723	0.783	**0.814**
6.80	8.52	8.27	8.05	7.92	0.747	0.784	0.817	**0.835**
6.80	8.13	7.79	7.73	7.98	0.804	0.855	**0.863**	0.826
7.10	7.61	7.23	7.13	7.04	0.929	0.981	**0.996**	0.991
7.30	7.82	7.41	7.79	7.99	0.928	**0.985**	0.932	0.906
7.90	8.22	8.32	8.29	8.29	**0.959**	0.947	0.950	0.951
8.60	7.94	8.41	10.2	10.5	0.923	**0.978**	0.815	0.778
9.20	8.89	9.30	9.58	8.99	0.966	**0.989**	0.958	0.977
10.1	9.89	10.1	9.78	9.61	0.979	**0.998**	0.968	0.951
10.6	7.97	9.21	10.2	9.14	0.752	0.869	**0.966**	0.862
10.7	10.5	10.0	10.5	10.7	0.981	0.939	0.980	**0.997**
11.1	10.3	10.1	9.84	9.54	**0.932**	0.910	0.886	0.859
11.2	13.0	11.9	11.6	11.4	0.843	0.942	0.963	**0.979**
11.2	10.6	10.4	10.3	10.1	**0.950**	0.929	0.916	0.903
12.5	11.5	11.3	11.4	11.5	**0.922**	0.908	0.910	0.917
13.8	15.7	14.5	14.4	14.6	0.860	0.953	**0.954**	0.940
14.3	13.2	13.0	13.4	13.1	0.926	0.909	**0.938**	0.918

*Note:* The highest “trueness” value is highlighted in bold.

Abbreviation: HAI = h-accuracy index.

## Data Availability

The data used to support the findings of this study are available from the corresponding author upon request.
